# Complement Factor H-Related Proteins CFHR2 and CFHR5 Represent Novel Ligands for the Infection-Associated CRASP Proteins of *Borrelia burgdorferi*

**DOI:** 10.1371/journal.pone.0013519

**Published:** 2010-10-20

**Authors:** Corinna Siegel, Teresia Hallström, Christine Skerka, Hannes Eberhardt, Barbara Uzonyi, Tobias Beckhaus, Michael Karas, Reinhard Wallich, Brian Stevenson, Peter F. Zipfel, Peter Kraiczy

**Affiliations:** 1 Institute of Medical Microbiology and Infection Control, University Hospital of Frankfurt, Frankfurt/Main, Germany; 2 Department of Infection Biology, Leibniz-Institute for Natural Products Research and Infection Biology, Jena, Germany; 3 Institute of Pharmaceutical Chemistry, Johann Wolfgang Goethe-University, Frankfurt/Main, Germany; 4 Institute of Immunology, University of Heidelberg, Heidelberg, Germany; 5 Department of Microbiology, Immunology and Molecular Genetics, University of Kentucky, Lexington, Kentucky, United States of America; 6 Friedrich Schiller University, Jena, Germany; National Institute of Allergy and Infectious Diseases, National Institutes of Health, United States of America

## Abstract

**Background:**

One virulence property of *Borrelia burgdorferi* is its resistance to innate immunity, in particular to complement-mediated killing. Serum-resistant *B. burgdorferi* express up to five distinct complement regulator-acquiring surface proteins (CRASP) which interact with complement regulator factor H (CFH) and factor H-like protein 1 (FHL1) or factor H-related protein 1 (CFHR1). In the present study we elucidate the role of the infection-associated CRASP-3 and CRASP-5 protein to serve as ligands for additional complement regulatory proteins as well as for complement resistance of *B. burgdorferi*.

**Methodology/Principal Findings:**

To elucidate whether CRASP-5 and CRASP-3 interact with various human proteins, both borrelial proteins were immobilized on magnetic beads. Following incubation with human serum, bound proteins were eluted and separated by Glycine-SDS-PAGE. In addition to CFH and CFHR1, complement regulators CFHR2 and CFHR5 were identified as novel ligands for both borrelial proteins by employing MALDI-TOF. To further assess the contributions of CRASP-3 and CRASP-5 to complement resistance, a serum-sensitive *B. garinii* strain G1 which lacks all CFH-binding proteins was used as a valuable model for functional analyses. Both CRASPs expressed on the *B. garinii* outer surface bound CFH as well as CFHR1 and CFHR2 in ELISA. In contrast, live *B. garinii* bound CFHR1, CFHR2, and CFHR5 and only miniscute amounts of CFH as demonstrated by serum adsorption assays and FACS analyses. Further functional analysis revealed that upon NHS incubation, CRASP-3 or CRASP-5 expressing borreliae were killed by complement.

**Conclusions/Significance:**

In the absence of CFH and the presence of CFHR1, CFHR2 and CFHR5, assembly and integration of the membrane attack complex was not efficiently inhibited indicating that CFH in co-operation with CFHR1, CFHR2 and CFHR5 supports complement evasion of *B. burgdorferi*.

## Introduction

Lyme disease is the most commonly reported vector-borne infectious disease in Eurasia and the United States. This multi-systemic inflammatory disease is caused by species of the *Borrelia burgdorferi* sensu lato complex, which includes *B. burgdorferi* sensu stricto (s.s.), *B. garinii*, and *B. afzelii* [1]. More recently, *B. spielmanii* has also been shown to be associated with cutaneous manifestations of Lyme disease [2]–[6]. Bacteria are transmitted to humans or other vertebrates through the bites of infected *Ixodes* spp. ticks. In most cases, human infection results in a localized skin rash accompanied by headache, myalgia, arthalgia, and fever, which usually resolve spontaneously. Untreated Lyme disease may lead to late manifestations that can include chronic arthritis, neurological abnormalities, cardiac complications, and skin lesions. The ability of Lyme disease borreliae to perpetuate their natural vertebrate-tick infectious cycle requires an array of strategies to survive in diverse host environments, and necessitates mechanisms to overcome innate and adaptive immune responses of several hosts. Lyme disease spirochetes are highly resistant to killing by the host's alternative pathway of complement [7], [8]. This is accomplished, at least in part, by the spirochetes camouflaging their outer surface with host-derived complement factor H (CFH) and factor H-like protein 1 (FHL1) which are fluid-phase immune regulators of the alternative complement pathway [9]–[12].

CFH and FHL1 are both encoded by the human CFH gene and are derived by alternative splicing [13]–[15]. The two proteins are structurally-related and fold into repetitive protein domains termed short consensus repeats (SCRs) [14], [15]. The SCRs, also termed as complement control protein modules are approximately 60 amino acids long and contain mainly beta-sheet structures which are stabilized by two conserved disulphide bridges. CFH is a 150 kDa glycoprotein that is composed of 20 SCR domains. FHL1 is a 42 kDa glycoprotein, comprised of the seven amino-terminal SCRs of CFH plus four unique amino acids at the C-terminus. Both CFH and FHL1 act as cofactors for factor I-mediated degradation of C3b and support the dissociation (decay-accelerating activity) of the C3 convertase, C3bBb [14]. The human CFH family also includes six “factor H-related” proteins (CFHR1, CFHR2, CFHR3, CFHR4A, CFHR4B, and CFHR5) [16], [17]. These proteins are all encoded by distinct genes, and individual domains show extensive sequence similarities to CFH [13]. The SCR domains toward the C-terminus of CFHR proteins share high degrees of similarity to the C-terminal surface binding region of SCRs 18–20 of CFH. This similarity suggests related and conserved function(s) [12]. The human CFHR1 protein consists of five SCRs and exists in two glycosylated forms, the 37-kDa CFHR1α and the 43-kDa CFHR1β protein [18], [19]. CFHR1 is a complement regulator that blocks C5 convertase activity as well as assembly and membrane insertion of the terminal components [20]. CFHR2 is composed of four SCRs and is found in plasma as a non-glycosylated 24-kDa form (CFHR2) and a glycosylated 29-kDa form (CFHR2α) [21]. The function(s) of CFHR2 is poorly understood. The 65-kDa CFHR5 protein is comprised of 9 SCRs and displays cofactor activity for factor I-mediated inactivation of C3b [16], [22]. CFHR5 also inhibited the activity of the fluid phase C3 convertase.

Lyme disease borreliae bind CFH, FHL1 and CFHR1 to their outer membranes through surface-exposed lipoproteins, collectively called “CRASPs” (*c*omplement *r*egulator-*a*cquiring *s*urface *p*roteins) [11], [23]–[27]. CRASPs are divided into three groups of genetically unrelated genes/proteins and different *B. burgdorferi* s.s. strains express different combinations of CRASP proteins. Each protein has different relative affinity for each of the three human immune regulators. Based on binding profile for CFH, FHL1 or CFHR1, the borrelial CRASPs expressed by *B. burgdorferi* s.s. are divided into (i) CFH and FHL1 binding proteins (BbCRASP-1 and BbCRASP-2), and (ii) molecules that interact with CFH and CFHR1, but not FHL1 (BbCRASP-3 to BbCRASP-5) [11], [23]–[25], [28]. BbCRASP-1, also termed CspA, is a member of the paralogous protein family 54 (PFam54), and is expressed by spirochetes only during tick-to-mammal and mammal-to-tick transmission stages, but not during persistent mammalian infection [26], [29]–[31]. The BbCRASP-2 molecule, which is also termed CspZ, is encoded by a unique gene and is expressed at high levels during mammalian infection [29], [32]. BbCRASP-3, BbCRASP-4 and BbCRASP-5, also known as ErpP, ErpC and ErpA, are closely-related members of the polymorphic *erp* gene family, and are expressed throughout mammalian infection [10], [27], [30], [33]–[37]. BbCRASP-3, BbCRASP-4, and BbCRASP-5 (hereafter referred to as CRASP-3, CRASP-4 and CRASP-5) bind CFH and CFHR1 through the C-terminal SCR(s), and do not bind the FHL1 protein. In contrast, BbCRASP-1 and BbCRASP-2 (hereafter referred to as CRASP-1 and CRASP-2) both bind to SCR-7 of CFH, which is shared with FHL1, enabling these two borrelial outer membrane proteins to bind both human complement regulators. Borrelial mutants lacking CRASP-1 and CRASP-2 are sensitive to complement-mediated killing in culture, and complementation with the CRASP-1 or CRASP-2 encoding genes (*cspA* or *cspZ*, respectively), facilitates survival in the presence of serum and, thus restores a serum-resistant phenotype [32], [38].

All investigated serum-resistant borrelial strains so far express the CFH/FHL1-binding CRASP-1 protein in combination with two or three of the CFH/CFHR1-binding, CRASP-3, CRASP-4 or CRASP-5 proteins. The potential of the single CFH/CFHR1-binding CRASP molecule for binding of additional members of the CFH protein family and the contribution of these CRASP proteins for complement resistance is still under debate. We first sought to identify whether recombinant CRASP-3 or CRASP-5 bind to additional serum proteins beside CFH, CFHR1, and plasminogen. Furthermore, we aimed to elucidate the role of these two borrelial proteins towards complement resistance by transformation of a serum-sensitive *B. garinii* strain which lacks CFH-binding CRASP proteins. The transformed borreliae were assayed for abilities to bind human serum proteins, surface deposition of complement activation products, and survival in the presence of normal human serum (NHS).

In the present study we identified two additional members of the human CFH protein family, CFHR2 and CFHR5 as novel ligands for CRASP-3 and CRASP-5 of *B. burgdorferi*. Binding of CFHR1, CFHR2, and CFHR5 to native and recombinant CRASP-3 and CRASP-5 proteins was more pronounced than that of CFH. The expression of either CRASP-3 or CRASP-5 in a serum-sensitive *B. garinii* strain lacking all CRASP proteins had no considerable effect on serum susceptibility suggesting that spirochetes must be able to acquire sufficient amounts of CFH on their surface beside CFHR1, CFHR2, and CFHR5 to survive in complement active serum.

## Results

### Identification of serum proteins that bind to CRASP-3 and CRASP-5

**Figure 1:**
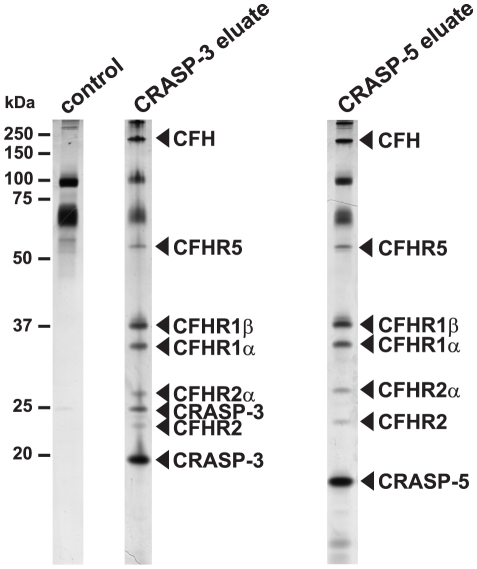
Identification of CRASP-3 and CRASP-5 ligands present in human serum. Recombinant, polyhistidine-tagged CRASP-3 or CRASP-5 proteins were immobilized onto magnetic beads and incubated with normal human serum. Empty beads were also incubated under the same conditions and used as a control to identify nonspecific binding of serum proteins. After extensive washing, bound proteins were eluted with 100 mM glycine-HCl and the eluate fractions were separated by Glycine-SDS-PAGE, following silver staining. Protein bands indicated were cut from stained gels and proteins were identified by mass spectrometry. Mobilities of molecular mass standards are indicated to the left.

To elucidate whether CRASP-3 and CRASP-5 bind several human proteins, the recombinant his-tagged CRASP-3 and CRASP-5 proteins were immobilized to magnetic beads. Following incubation with NHS, beads were extensively washed and the recombinant proteins along with bound serum proteins were eluted. Eluates were separated by Glycine-SDS-PAGE and analyzed by silver staining (Fig. 1). In the elute fraction of CRASP-3- and CRASP-5-coupled beads, proteins with apparent molecular mass of 180-, 55-, 37-, 32-, 29- and 24-kDa were detected. Two additional proteins of 25- and 20-kDa were found in the elute fraction of CRASP-3-coupled beads, while an 18-kDa protein was detected only in the elute fraction of CRASP-5-coupled beads. Several proteins in the 60- to 80-kDa range that also attach to uncoated beads were excluded from further analysis. A very faint band of 25-kDa could also be found in the control lane. All eluted proteins were subjected to in-gel trypsin digestion and peptides were analyzed using MALDI-TOF. Obtained spectra were searched against the NCBI.fasta protein database and a score >80 was defined to be significant (p<0.05). The 180-kDa band found in both elution fractions yield a high protein score of >300 of a number of the tryptic peptides representing the complement regulator CFH. Peptides of the 55-kDa protein were identified as fragments of CFHR5 and tryptic peptides of the 37- and 32-kDa protein represented CFHR1β and CFHR1α, the two different glycosylated forms of CFHR1 [39]. The 29- and the 24-kDa band were identified as CFHR2α and CFHR2, respectively. The 25-kDa and 20-kDa bands in the eluate fraction of CRASP-3-coupled beads corresponded to CRASP-3 itself. Likewise, the 18-kDa band in the eluate fraction of CRASP-5-coupled beads was identified as CRASP-5. Thus, CRASP-3 and CRASP-5 bind several members of the human CFH protein family including CFHR1, CFHR2, and CFHR5.

### Binding of human serum proteins CFH, CFHR1, CFHR2 and CFHR5 to CRASP-3 and CRASP-5

**Figure 2:**
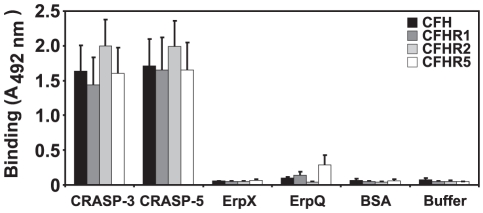
CRASP-3 and CRASP-5 bind diverse complement proteins. Binding of equimolar amounts of CFH, CFHR1, CFHR2, CFHR5 (33 µM) to immobilized CRASP-3, CRASP-5, ErpX, and ErpQ (5 µg/ml) was analyzed by ELISA. Bound CFH or CFHR proteins were detected with either goat CFH polyclonal antiserum or mouse CFHR1 monoclonal antiserum (JHD 7.10), which reacts with all the three CFHRs. Data represent the means and standard errors from three separate experiments.

Next, binding of recombinant CRASP-3 and CRASP-5 to each of the three human identified serum proteins was analyzed by ELISA (Fig. 2). CRASP-3 and CRASP-5 as well as ErpX and ErpQ, two outer surface proteins which belong to the paralogous Erp protein family and included as controls were immobilized onto microtiter plates and incubated separately with purified recombinant CFHR1, CFHR2 and CFHR5. Purified CFH served as a control. All three CFHR proteins bound to both CRASP-3 and CRASP-5 but not to ErpX or ErpQ.

### Ectopic expression of CRASP-3 and CRASP-5 in CRASP-deficient *B. garinii*

**Figure 3:**
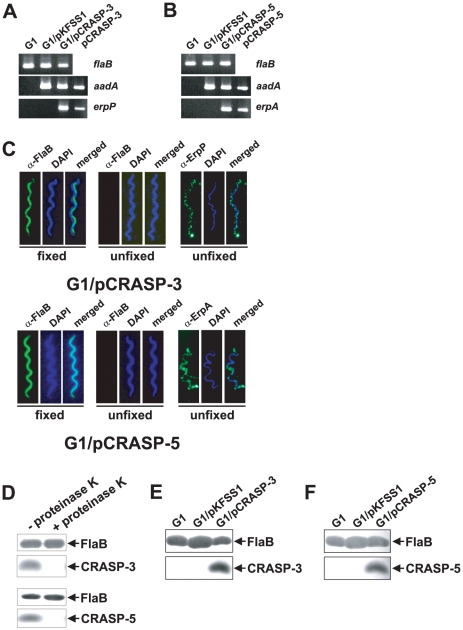
Characterization of *B. garinii* G1 transformants producing CRASP-3 or CRASP-5. (A and B) *B. garinii* G1 and transformed G1 strains were characterized by PCR amplification using *flaB*, *aadA*, *erpP*, and *erpA* gene-specific primers, as listed in Table 1. Both panels, left to right: wild-type *B. garinii* G1, *B. garinii* G1 transformed with the empty cloning vector pKFSS1, *B. garinii* G1 transformed with either pCRASP-3 or pCRASP-5, and purified pCRASP-3 or pCRASP-5 alone. (C) Demonstration of surface expression of CRASP-3 and CRASP-5 by transformed *B. garinii* G1, by indirect immunofluoresecence microscopy of intact borrelial cells. Spirochetes were incubated with rabbit polyclonal anti-CRASP-3 or anti-CRASP-5 antisera before fixation. Periplasmic FlaB used as control was detected by mAb L41 1C11 using fixed and unfixed cells. For counterstaining, the DNA-binding dye DAPI were used to identify all bacteria. Slides were visualized at a magnification of ×1000 using a Olympus CX40 fluorescence microscope mounted with a DS-5Mc charge-coupled device camera (Nikon). (D) Surface localization of CRASP-3 and CRASP-5 in transformed G1/pCRASP-3 and G1/pCRASP-5. Spirochetes were incubated with or without proteinase K, then lysed by sonication and total proteins separated by Tricine-SDS-PAGE. CRASP-3 and CRASP-5 were identified using NHS and MAb VIG8 specific for the C-terminus of CFH by ligand affinity analysis. Flagellin (FlaB) was detected with MAb L41 1C11 (dilution 1/1000) by Western blotting. (E and F) Synthesis of CRASP-3 (panel D) and CRASP-5 (panel E) by transformed G1 as assessed by ligand-affinity blotting. Whole cell lysates were separated by Tricine-SDS-PAGE and transferred to nitrocellulose. The membranes were incubated with NHS and binding of CFH to borrelial proteins was detected with mAb VIG8. Monoclonal antibody, L41 1C11, specific for the flagellin protein FlaB, was used to show equal loading of bacterial lysates.

While serum-resistant isolates of *B. burgdorferi* may express up to five distinct CFH-binding CRASP molecules, the serum-sensitive *B. garinii* strain G1 does not express any of these proteins during laboratory cultivation [23]. Thus, *B. garinii* G1 represents a useful model for functional analyses of the individual CRASP proteins. *B. garinii* G1 was transformed with plasmids pCRASP-3 or pCRASP-5 as well as with the empty shuttle plasmid pKFSS1. Transformations were confirmed by PCR amplification of the plasmids' streptomycin resistance gene (*aadA*) and the CRASP-3 encoding *erpP* or CRASP-5 encoding *erpA* gene (Fig. 3A). Strains G1/pCRASP-3 and G1/pCRASP-5 each yielded an amplicon that correspond to *erpP* or *erpA*, whereas the control strains G1 and G1/pKFSS1 did not. The streptomycin resistance gene of the recombinant plasmid was detected in all transformed, but not in the untransformed wild-type strain G1.

CRASP-3 and CRASP-5 are surface-exposed, outer membrane proteins of *B. burgdorferi* [40]. To confirm surface-exposure of these proteins in transformed *B. garinii*, immunofluorescence microscopy was conducted using live bacteria and polyclonal antibodies specific for either CRASP-3 or CRASP-5 [40]. To avoid damage to the fragile borrelial outer membrane, intact bacteria were incubated with antibodies before being fixed onto glass slides and sealed with mounting medium containing the DNA-binding dye DAPI. Both CRASP-3 of transformed strain G1/pCRASP-3 and CRASP-5 of strain G1/pCRASP-5 were detected on the outer membrane (Fig. 3C). Intactness of the fragile borrelial outer membrane was confirmed by demonstrating lack of binding of antibodies directed against the periplasmic flagellar protein FlaB (Fig. 3C middle panels). In contrast, permeabilized spirochetes showed a strong staining with the same antibody (Fig. 3C, left panels). Control strains G1 or G1/pKFSS1 lack any fluorescence reactivity with each antiserum (data not shown). Surface localization of CRASP-3 and CRASP-5 was examined, by incubation of intact bacteria with proteinase K, followed by ligand affinity blot analyses of borrelial lysates. Ligand affinity blot analyses of protease-treated cells revealed that CRASP-3 and CRASP-5 were susceptible to proteinase K digestion (Fig. 3D).

CFH-binding to borreliae was also analyzed for cell lysates derived from CRASP-3 and CRASP-5 expressing transformants and the non-expressing control strains G1 and G1/pKFSS1. Ligand affinity blot demonstrated binding of CFH to G1/pCRASP-3 and G1/pCRASP-5 but not G1 and G1/pKFSS1 (Fig. 3E and F, respectively). Thus, CRASP-3 and CRASP-5 exposed on the borrelial outer surface binds human CFH. This allowed further assays for the interaction of CRASP-3 and CRASP-5 with the CFHRs serum proteins.

### Binding of human serum proteins by transformed *B. garinii*

**Figure 4:**
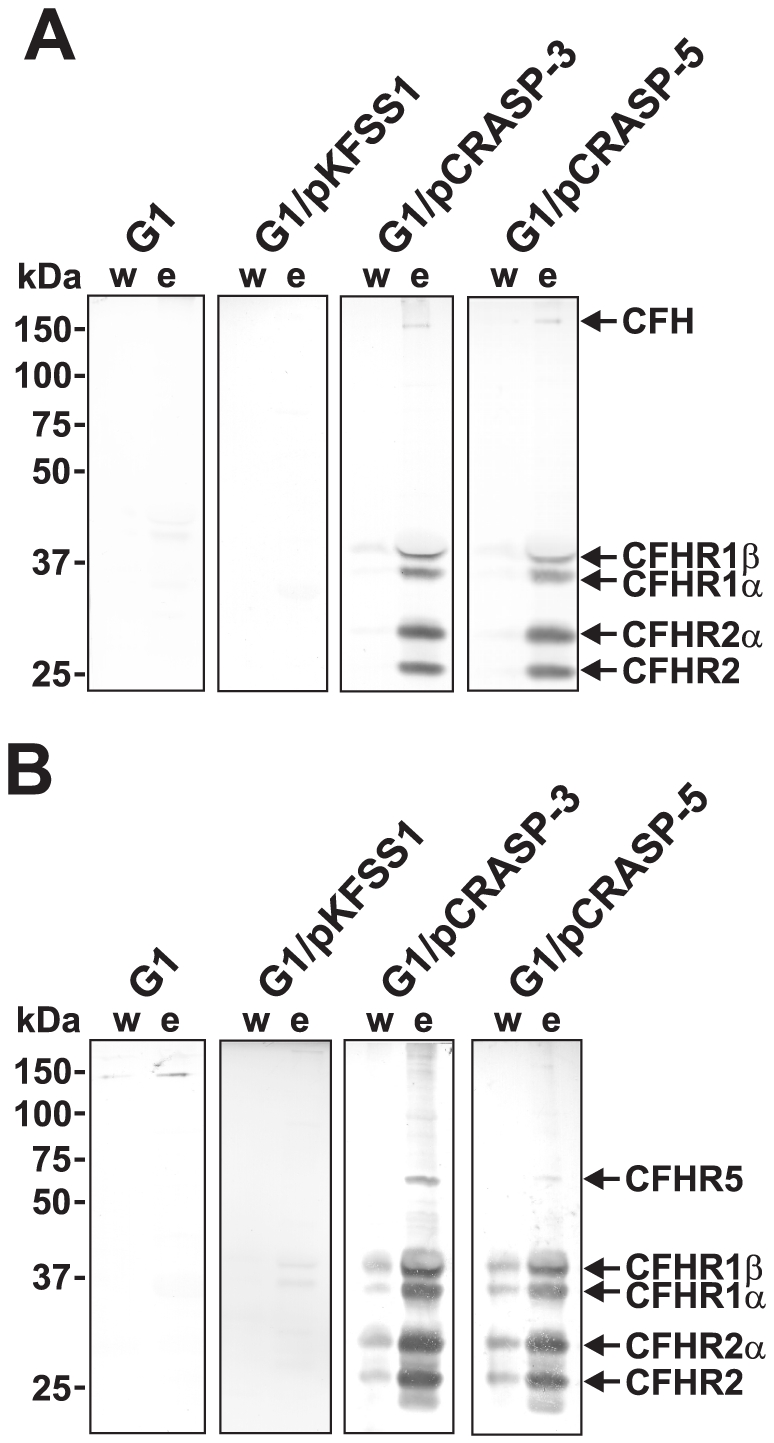
Binding of serum molecules by *B. garinii* transformants. *B. garinii* strains G1, G1/pKFSS1, G1/pCRASP-3, and G1/pCRASP-5 were incubated in NHS plus EDTA to prevent complement activation, washed extensively, and then bound proteins were eluted using 0.1 M glycine (pH 2.0). Both the last wash (w) and the eluate (e) fractions obtained from each strain were separated by Glycine-SDS-PAGE and transferred to nitrocellulose. Membranes were probed with either (A) MAb VIG8, which recognizes the C-terminus of CFH and CFHR proteins, or (B) mAb JHD 7.10, which recognizes CFHR1 and CFHR2, but not CFH. Probable identities of protein bands (confirmed by data shown in Fig. 1) are indicated to the right of each panel. Mobilities of molecular mass standards are shown to the left of the panels.

We next examined whether transformed strains G1/pCRASP-3 and G1/pCRASP-5 bind human complement regulators. Spirochetes incubated in EDTA-treated NHS were washed extensively and bound proteins were eluted. The final wash and the elute fraction were separated by Glycine-SDS-PAGE and after transfer to nitrocellulose, presence of CFH was analyzed by immunoblot with a specific mAb. CRASP-3 and CRASP-5 expressing transformants bound low amounts of CFH (Fig. 4A). In addition, four prominent bands with an mobility or apparent mass of 43-, 37-, 29- and 24-kDa were present in elute fractions of G1/pCRASP-3 and G1/pCRASP-5. In contrast, wild-type strain G1 and transformant G1/pKFSS1 did not bind CFH and CFH-related proteins. Based on the reactivity with the mAb VIG8 which reacts with CFH, CFHR1 and CFHR2 [41] and based on the mobility, the 43- and 37-kDa proteins correspond the two glycosylated forms CFHR1α and CFHR1β. Similarly, the 29- and 24-kDa bands represent the non-glycosylated and the glycosylated form of CFHR2.

The identity of the bands was confirmed with mAb JHD 7.10, which is specific for the N-terminus of CFHR1, CFHR2, and CFHR5 but which does not react with CFH or FHL1 [42]. The mAB JHD 7.10 identified bands of 50-, 43-, 37-, 29-, and 24-kDa which represent CFHR1, CFHR2, and CFHR5, respectively (Fig. 4B). Thus, CRASP-3 and CRASP-5 expressed on the surface of live borreliae bind the human serum proteins CFHR1 and CFHR2, CFHR5, and only miniscute amounts of CFH.

### Serum proteins bound with different intensities to transformed borrelial cells

**Figure 5:**
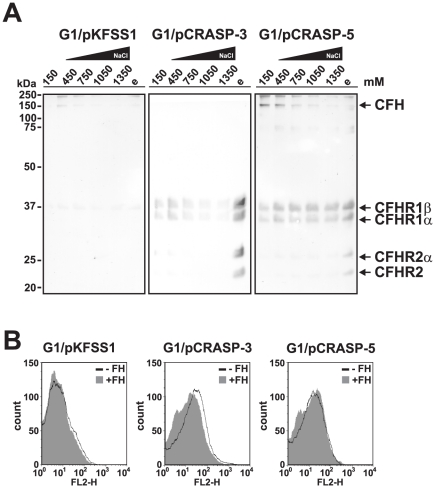
Serum proteins display differential binding capabilities to CRASP-3 and CRASP-5. (A) The binding capabilites of serum proteins to *B. garinii* strains G1/pKFSS1, G1/pCRASP-3, and G1/pCRASP-5 were assessed in the presence of increasing salt concentrations. Spirochetes were incubated in NHS plus EDTA, washed fourfold with PBSA containing 0.05% Tween20. Cells were then resuspended in PBSA containing 450 mM NaCl, incubated for 15 min at room temperature, and sedimented by centrifugation. The steps were repeated with increasing concentrations of NaCl. Strong binding proteins were finally eluted using 0.1 M glycine buffer (e). The supernatants obtained from the last wash fraction (150 mM NaCl), fractions from the incubation reactions (450, 750, 1050, 1350 mM NaCl), and the eluate fraction were then separated by Glycine-SDS-PAGE and transferred to nitrocellulose. Membranes were probed with polyclonal anti-CFHR1 antiserum to detect CFH and CFHR proteins. Mobilities of molecular mass standards are shown to the left of the panels. (B) The binding capability of CFH to G1/pCRASP-3 and G1/pCRASP-5 was further analyzed by flow cytometry. The binding of CFH to G1/pCRASP-3 and G1/pCRASP-5 is shown by the solid line while the grey shaded histogramm represents the binding of control strain G1/pKFSS1 (control). Borrelial cells were incubated with 4 µg CFH. The x-acis shows the fluorescence on a log_10_ scale and the the y-acis represents the numbers of events. The isotype control (no CFH added) has been omitted for easier visualization.

To define the effect of NaCl on the interaction of the human serum proteins to CRASP expressed on transformed borrelial strains G1/pCRASP-3, G1/pCRASP-5 and G1/pKFSS1 was assayed. To this end spirochetes were washed extensively with a buffer containing 150 mM NaCl and cell-bound proteins were subsequently eluted with increasing concentrations of salt (450 mM to 1350 mM). The elute fractions were then separated by Glycine-SDS-PAGE and after transfer to nitrocellulose, human serum proteins were detected with a polyclonal anti-CFHR1 antiserum (Fig. 5A). Concerning G1/pCRASP-3, neither CFH nor CFHR2 and CFHR5 could be detected in the fractions containing increasing salt concentrations (Fig. 5A, middle panel). In contrast, CFHR1 and CFHR2 as well as their glycosylated forms were readily detectable in the final glycine fraction suggesting that these serum proteins possess a stronger binding capacity to surface-exposed CRASP-3. As depicted in Figure 5A (right panel), the faint band of 150-kDa corresponding to CFH detected after incubation of G1/pCRASP-5 with up to 450 mM NaCl and which signal decreased at higher salt concentrations, suggests that CFH is relatively loosely attached to the borrelial surface. The 150-kDa band was also hardly visible in the respective fractions of the control strain G1/pKFSS1, thus argueing for a non-specific binding of CFH to the borrelial surface. In contrast, CFHR1 was detectable in all fractions of transformants expressing CRASP-3 or CRASP-5 even in the highest concentration of NaCl as well as in the final glycine fraction. CFHR2 was only detected in the final glycine fraction, suggesting a stronger binding capacity of CFHR2 to CRASP-3 and CRASP-5. However, CFHR5 was not detected by this assay. No CFHRs proteins were identified in the control transformed strain G1/pKFSS1 (Fig. 5A, left panel).

Employing flow cytometry for analyzing binding of CFH to G1/pKFSS1, G1/pCRASP-3, and G1/pCRASP-5, this complement regulator was bound to some extent to the surface of the CRASP expressing strains but not to the control strain (Fig. 5B). Taken together, binding of CFHR1 and CFHR2 to borrelial cells expressing CRASP-3 and CRASP-5 was more pronounced than that of CFH and CFHR5.

### Serum susceptibility of *B. garinii* producing CRASP-3 or CRASP-5

**Figure 6:**
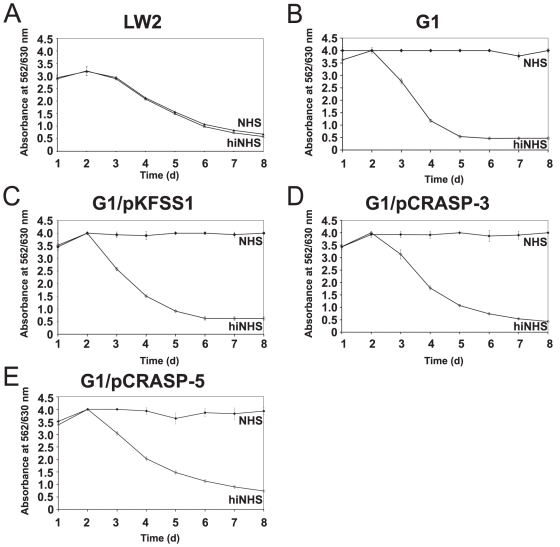
Serum susceptibility of transformed *B. garinii* G1. A growth inhibition assay was used to investigate susceptibility to human serum of *B. burgdorferi* s.s. strain LW2 (A), and *B. garinii* strains G1 (B), G1/pKFSS1 (C), G1/pCRASP-3 (D), and G1/pCRASP-5 (E). Spirochetes were incubated in either 50% NHS (filled diamonds) or 50% heat-inactivated NHS (open diamonds) over a cultivation period of 8 days at 33°C, respectively. Color changes were monitored by measurement of the absorbance at 562/630 nm. All experiments were performed three times in which each test was done at least threefold with very similar results. For clarity only data from representative experiments are shown. Error bars represent ± SD.

Having demonstrated binding of CFHRs to intact borrelial cells, the role of CFHR for complement resistance was assayed under more physiological conditions. *B. garinii* strain G1 is sensitive to complement and does not survive in NHS while wild-type *B. burgdorferi* LW2 resist complement-mediated killing and survives even in high concentrations of NHS [7], [43]. Therefore, survival and growth of CRASP-3 and CRASP-5 expressing spirochetes in NHS was assayed. Neither of the CRASP-3 or CRASP-5 expressing transformants grew in the presence of NHS (Fig. 6D and E) suggesting that binding of CFHR1, CFHR2, and CFHR5 alone is not sufficient for complement resistance. The serum-resistant strain LW2 grew equally well in medium supplemented with NHS or heat-inactivated NHS (Fig. 6A) while both isolates G1 and G1/pKFSS1 survived only when heat-inactivated NHS was used (Fig. 6B and C).

**Figure 7:**
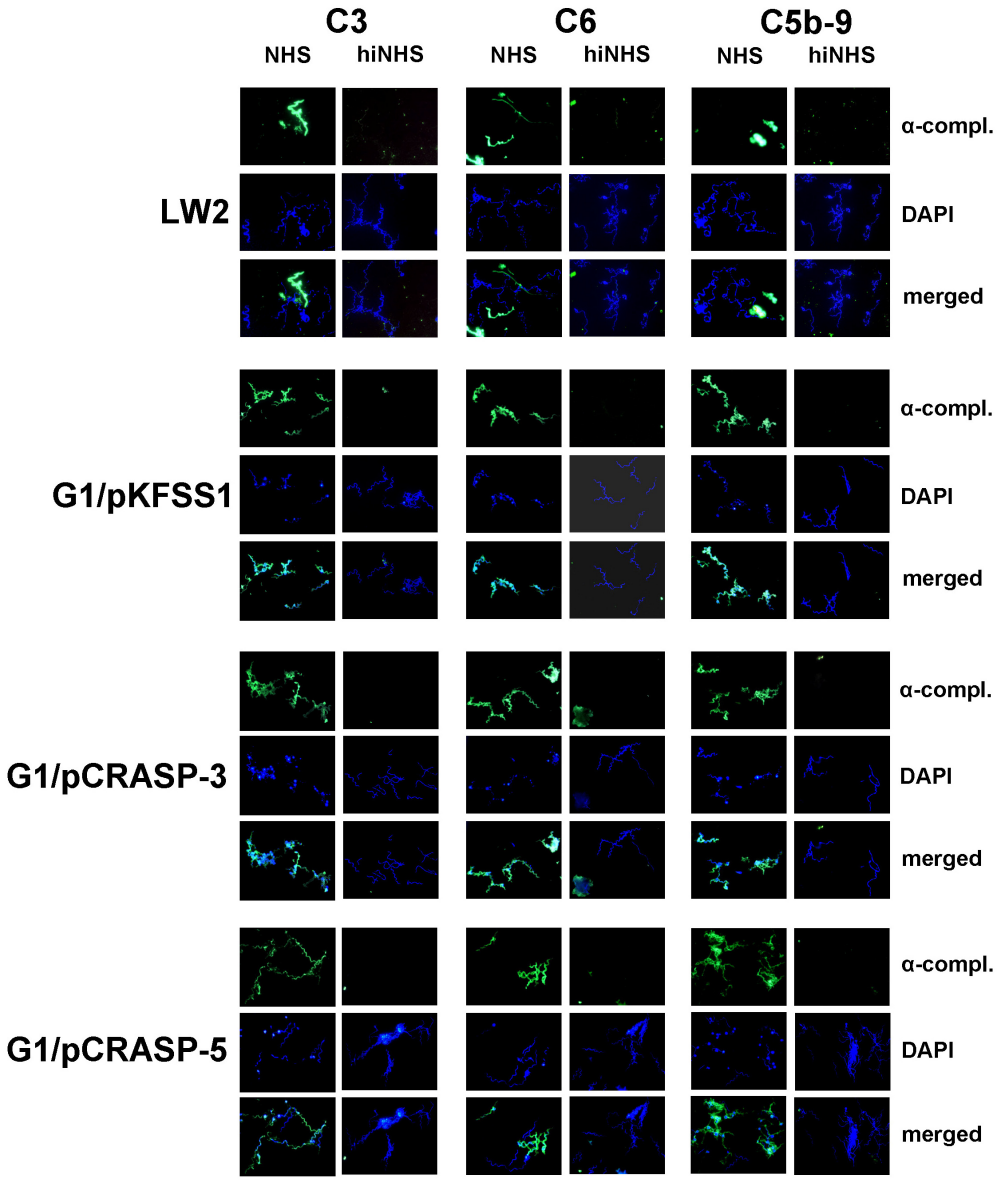
Deposition of complement components C3, C6 and C5b-9 on the surface of *B. garinii* G1 producing CRASP-3 or CRASP-5. Complement components deposited on *B. burgdorferi* s.s. LW2 (control strain), transformants G1/pKFSS1, G1/pCRASP-3, and G1/pCRASP-5 were detected by indirect immunofluorescence microscopy. Spirochetes were incubated with either 25% normal human serum (NHS) or heat-inactivated NHS (hiNHS). Bound C3, C6, or C5b-9 were detected using specific antibodies against each component plus appropriate Alexa 488-conjugated secondary antibodies. For visualization of intact spirochetes, the DNA-binding dye DAPI was used. Slides were visualized at a magnification of ×1000 and the data were recorded via a DS-5Mc CCD camera (Nikon) mounted on an Olympus CX40 fluorescence microscope. Panels shown are representative of at least 20 microscope fields.

Next deposition of complement activation products was analyzed on the bacterial surface. Following incubation in NHS, the two transformed strains G1/pCRASP-3 and G1/pCRASP-5 as well as G1/pKFSS1 and the wild-type strain G1 showed surface deposition of C3, C6 and C5b-9 (Fig. 7). Furthermore, extensive bleb formation and lack of DAPI staining suggests that cells are lysed. In contrast, bacteria incubated with heat-inactivated NHS did not show evidence of complement deposition.

To exclude that misfolding of the exogenously expressed CRASP-3 and CRASP-5 by *B. garinii*, similar analyses were performed in a *B. burgdorferi* mutant strain, B313. This derivative of strain B31 expresses only CRASP-5 and lacks all other CRASP-encoding genes. Again strain B313 was transformed with pCRASP-3 and pCRASP-5 did not grow in the presence of 50% NHS (data not shown).

Taken together, binding of CFHRs by CRASP-3 or CRASP-5 producing borreliae does not protect spirochetes from complement-mediated killing.

### Detection of C3b inactivation products after incubation of *Borrelia* with CFH

**Figure 8:**
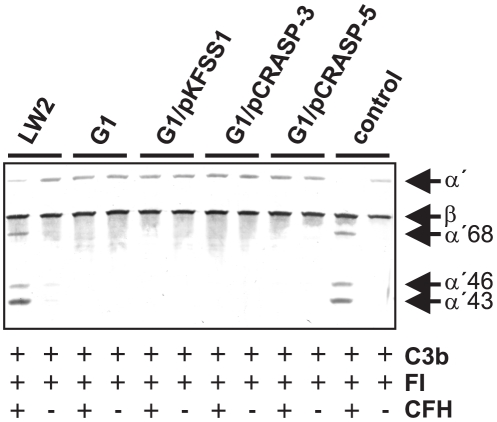
Detection of C3b inactivation products after incubation of CFH with *B. garinii* transformants. Factor I-mediated conversion of C3b to iC3b was analyzed by detection of C3b cleavage fragments after incubation of spirochetes with purified CFH. *B. burgdorferi* s.s. LW2 producing all five CRASP proteins (control strain), *B. garinii* G1, G1/pKFSS1, G1/pCRASP-3, and G1/pCRASP-5 were incubated with CFH for 60 min at room temperature. After extensive washing with PBS, C3b (10 ng/ml) and factor I (20 ng/ml) were added and the mixture was incubated for 30 min at 37°C. For control purposes all reactions were also performed in the absence of CFH. Subsequently, the probes were boiled for 5 min, subjected to 12.5% Glycine-SDS-PAGE and transferred onto a nitrocellulose membrane. The various C3b degradation products (α'46- and α'43-kDa bands) were visualized by Western blotting using a polyclonal goat anti-human C3 antiserum (Calbiochem). As additional controls, reaction mixtures containing C3b and factor I were incubated with or without purified CFH (lanes 11 and 12), respectively.

As CFH is loosely bound to CRASP-3- and CRASP-5 positive cells, we aimed to analyze if this residual CFH can inhibit alternative pathway activity and promote factor I-mediated C3b inactivation. Transformed borreliae G1/pCRASP-3 and G1/pCRASP-5, as well as G1/pKFSS1, *B. garinii* G1 and *B. burgdorferi* s.s. LW2 (control) were first incubated with purified CFH, and after washing factor I and C3b were added. Following incubation for 60 min, aliquots were subjected to Glycine-SDS-PAGE, and C3b cleavage products were detected by Western blotting. CFH retained cofactor activity when bound to *B. burgdorferi* s.s. LW2 as demonstrated by the appearance of C3b inactivation products α'68, α'46 and α'43 kDa (Fig. 8). In contrast, none of the *B. garinii* strains preincubated with CFH promoted cleavage of C3b in the presence of factor I, suggesting that CFH was unable to maintain its regulatory activity or extremely low amounts of C3 cleavage products were generated.

## Discussion

*B. burgdorferi* employs multiple strategies to survive in and persistent in the human host. One central immune evasion strategy is the ability of borreliae to camouflage their surface with host fluid phase complement regulators, CFH and FHL1 [10]–[12], [33]. This strategy allows the spirochetes to control, inhibit and finely regulate complement activation directly on the surface. Here, we extend the characterization of the infection-associated CRASP proteins, CRASP-3 and CRASP-5 of *B. burgdorferi* s.s. and show that these two molecules bind the host immune regulators CFHR2 and CFHR5. To our knowledge, this is the first report which demonstrates binding of CFHR2 and CFHR5 to a human pathogen. Native CRASP-3 and CRASP-5 also bind complement regulator CFHR1 and both isoforms of CFHR1 and CFHR2. For CFHR2 the CRASP-3 and CRASP-5 binding domain is likely also located in the C-terminus as SCR4 of CFHR2 shows a high level of sequence identity (89% on the amino acid level) to SCR19 of CFH [13].

Purified, recombinant CRASP-3, CRASP-4, CRASP-5, and other, closely related Erp proteins such as OspE and p21 derived from different *B. burgdorferi* s.s. isolates bind human CFH [10], [27], [33], [34], [44]–[47]. Here we demonstrate that CRASP-3 and CRASP-5 expressed by genetically engineered *B. garinii* bind CFH in solid-phase affinity blot experiments. However, the intact transformed *B. garinii*, bind CFHR1, CFHR2 and CFHR5 and bind CFH from human serum with low affinity (compared Fig. 4 and 5). This difference may be due the lack of two additional distinct CFH-binding proteins, CRASP-1 and CRASP-2 in *B. garinii* strains. Serum CFH has a coiled, folded-back conformation, with the C-terminus extended and at physiological concentrations can form dimeric or oligomeric complexes in solution [48]–[52]. Binding of CFH to human cells is initially mediated by the C-terminal domains SCR19 and 20, which then leads to an uncoiling of the protein and exposure of other CFH domains to additional cell-surface receptors [13], [53]–[55]. The various CRASPs may play similar roles, with initial interaction of the C-terminus of CFH by CRASP-3 or CRASP-5 followed by binding to internal regions SCR5 to 7 of CFH by CRASP-1 or CRASP-2. Conceivably, the writhing of live spirochetes may dislodge long, extended and weakly bound CFH molecules from the bacterial surface, whereas the much smaller serum proteins CFHR1, CFHR2, and CFHR5 are more likely to be retained. In addition, the stronger affinity of CRASP-3 and CRASP-5 to CFHR1 and CFHR2 compare to CFH may also favor preferred binding of these molecules to borrelial cells, even though CFH is present in a 10-fold higher concentration in plasma than CFHR1, CFHR2 or CFHR5 [28], [56]. Furthermore, CFHR1 which lacks C3-mediated cofactor activity but inhibits C5 convertase activity and MAC assembly might compete under physiological conditions with CFH for CRASP-3 and CRASP-5 binding (CFHR1:CFH ratio of 0.37:1) and thus reduce local CFH-mediated complement regulatory activity [28], [56]. Displacement of CFH by CFHR1 (using equal molar amounts of both proteins) is accompanied with a significant reduction of C3b degradation products as recently demonstrated by Heinen et al. [20]. Moreover, CFH improperly bound to the borrelial surface appears to be unable to efficiently inactivate deposited C3b or accelerate the decay of the formed C3 convertase after activation of the complement cascade. This might explains why no C3b cleavage products were detectable when *B. garinii* producing CRASP-3 or CRASP-5 were incubated with purified CFH or human serum (Fig. 8). It is likely that CFHR2, which exhibits sequence identities of 89% and 61% to the C-terminal SCRs 19 and 20 of CFH, respectively, also competes with CFH for the same binding site(s) on the two borrelial proteins.

Apparently as a consequence of the inability of CFH to bind to the microbial surface, it appears that the interactions between CFHR1, CFHR2 and CFHR5 and CRASP-3 and CRASP-5 proteins are not adequate to sufficiently protect borreliae against complement-mediated killing (Fig. 6). This may explain why these bacteria accumulated lethal complement activation products [C3, C6 and C5b-9 (MAC)] on their surfaces (Fig. 7). Once complement is activated by the borreliae, it seems that the inhibitory activity of CFHR1 on the C5 convertase and the cofactor activity for factor I-mediated inactivation of C3b by CFHR5 can not completely retard formation and insertion of the MAC pointing to an crucial role of human CFH in complement resistance of borreliae. However, preincubation of borreliae with purified CFHR1 before treatment of the spirochetes with complement active CFH-depleted serum did not enhance survival of the transformants indicating that CFHR1 alone cannot restore the complement inhibitory function of CFH (data not shown). Thus, human CFH plays a major role in evading complement attack of *B. burgdorferi*.

CFHR1 and CFHR5 and likely also CFHR2 have complement-regulatory activities, thus surface recruitment of these host proteins may help *Borrelia* to control complement activation. Apparently the three CFHR proteins alone are not sufficient to control complement activation at the surface of *Borrelia*. Most likely the CFHR proteins cooperate with CFH. CFHR1 and CFHR2 are major constituents of serum lipoprotein particles that also contain phospholipids, apolipoprotein A-I, lipopolysaccharide-binding protein, and fibrinogen [57], [58]. Thus, it is possible that Lyme disease borreliae capture CFHRs or lipoprotein particles to allow adherence to host epithelial cells and tissues, as recently described for CFH-coated *S. pneumoniae* [59].

Brooks et al. reported that a B31 mutant strain carrying two native copies of the *erpA* gene, but lacking the CRASP-1 encoding *cspA*, displayed a serum-sensitive phenotype [60]. That result hinted that expression of only CRASP-3 and CRASP-5 does not sufficiently protect Lyme disease borreliae from complement-mediated killing. However, in another report, when *B. garinii* strain 50/97 was genetically modified to produce the OspE protein of *B. burgdorferi* s.s. strain 297 (a homolog of strain B31 CRASP-3 and CRASP-5), those bacteria survived in the presence of 40% NHS for up to 5 h [61]. That latter result may indicate that the strain 297 OspE protein is functionally different from the paralogous CRASP-3 and CRASP-5 proteins of strain B31. Alternatively, Alitalo et al. did not report having examined *B. garinii* strain 50/97 to see if it produced a CFH-binding protein(s) homologous to CRASP-1 or CRASP-2 [61]. It is possible that, if strain 50/97 produced a CRASP-1 or CRASP-2 homolog, the *ospE* transformant would express both types of CFH-binding proteins on its surface, and thereby bind CFH at levels sufficient to prevent complement-mediated killing. Note that the current study used *B. garinii* G1 and *B. burgdorferi* s.s. B313, both of which have been demonstrated to lack CRASP-1 and CRASP-2 homologs. Additional analyses of *B. garinii* strain 50/97, as well as production and examination of transformed bacteria that specifically express a CRASP-3/CRASP-5/OspE protein plus CRASP-1 or CRASP-2 will help clarify each CRASP alone and in combination.

In summary, we identified two new ligands, CFHR2 and CFHR5 for the infection-associated CRASP-3 and CRASP-5 proteins of *B. burgdorferi*. In the absence of CRASP-1 and CRASP-2, live borreliae that express CRASP-3 or CRASP-5 preferentially bind CFHR1, CFHR2 and CFHR5 while binding of CFH was rather weak. The capability of *B. burgdorferi* to interact with different members of the CFH protein family via distinct CRASPs suggests a role of these CFHRs in immune evasion of this particular pathogen.

## Materials and Methods

### Ethics statement

The study and the respective consent documents were approved by the ethic committee at the Johann Wolfgang Goethe-University of Frankfurt (control number 160/10). All healthy blood donors provided written informed consent.

### Bacterial strains and culture conditions

*B. burgdorferi* s.s. strains B31 (tick isolate, USA) and LW2 (skin isolate, Germany), *B. garinii* isolate G1 (CSF isolate, Germany) classified by RFLP analysis as OspA serotype 6 according to Michel et al. [4], and *B. garinii* transformants G1/pKFSS1, G1/pCRASP-3 and G1/pCRASP-5 were grown at 33°C for 2 to 4 days to mid-exponential phase (1×10^7^ to 5×10^7^ spirochetes/ml) in modified Barbour-Stoenner-Kelly (BSK) medium [23] or BSK supplemented with streptomycin at a final concentration of 20 µg/ml. The density of spirochetes was determined using dark-field microscopy and a Kova counting chamber (Hycor Biomedical, Garden Grove, CA). *Escherichia coli* DH5α or JM109 used for cloning experiments and protein expression were grown at 37°C in yeast tryptone supplemented with appropriate antibiotics.

### Human sera, polyclonal and monoclonal antibodies

Non-immune human serum (NHS) obtained from 20 healthy human blood donors without known history of spirochetal infections was used as source for CFH. Sera that proved negative for anti-borrelial antibodies were combined to form the NHS pool. For some studies, human serum samples were incubated at 56°C for 30 min to inactivate complement.

A goat polyclonal antiserum was used to detect human CFH (Merck Biosciences, Bad Soden, Germany and Complement Technology, Tyler, TE). Mouse polyclonal anti-CFHR1 antibody or mAb JHD 7.10 were used for detection of CFHR1 and CFHR2 and CFHR5 [41]. The goat anti-human C3 and C6 antibodies were purchased from Calbiochem, and the monoclonal anti-human C5b-9 antibody was obtained from Quidel (San Diego, CA, USA). To detect borrelial proteins specific MAb and polyclonal antibodies were used as follows: MAb L41 1C11 was used to recognize the flagellar component FlaB, MAb N38 1.1 and rabbit polyclonal antiserum αCRASP-3 was used to identify CRASP-3, and MAb B11 and rabbit polyclonal antiserum αCRASP-5 were used to detect CRASP-5 [27], [62], [63].

### Expression of recombinant borrelial proteins

Generation of vectors pBLS538, pBLS527, pBLS536, and pBLS539 producing amino-terminally polyhistidine-tagged CRASP-3(ErpP), CRASP-5(ErpA), ErpQ, and ErpX, respectively were described previously [64]. Note that the CRASP-3 and CRASP-5 encoded by *erpP* and *erpA* of *B. burgdorferi* s.s. type strain B31 and the European *B. burgdorferi* s.s. strain LW2 are identical.

Expression of recombinant proteins was induced in DH5α at an OD_600_ of 0.6 by the addition of 0.2 mM IPTG. Following incubation for 4 h at room temperature, cells were centrifuged (5000×g, 20 min, 4°C) and subsequently suspended in lysis buffer (300 mM NaCl, 56 mM NaH_2_PO_4_ pH 8, 10 mM imidazole) containing 50 mg/ml lysozyme. Cells were lysed by 6 rounds of sonication for 30 sec using a Branson B-12 sonifier (Heinemann, Schwäbisch Gmünd, Germany). After centrifugation (14,000×g, 20 min, 4°C), supernatants were filtered through 0.45 µm filters and stored at −20°C until used.

### Expression of recombinant CFHR1, CFHR2, and CFHR5

Recombinant FHL1 and CFHR1 were expressed in *Spodoptera frugiperda* Sf9 insect cells infected with recombinant baculovirus. The cloning of various deletion constructs, expression, and purification has been described previously [28], [56], [65], [66].

The full length CFHR2 cDNA was cloned into pPICZαB (Invitrogen) and the protein was expressed in the yeast *Pichia pastoris* strain X33 according to standard protocols (Eberhardt et al, manuscript in preparation). The full length CFHR5 cDNA was cloned into pBSV-8His and expressed in the baculovirus system as described [66] Uzonyi et al., manuscript in preparation]. All expressed His-tagged recombinant proteins were purified by Ni^2+^ chelate affinity chromatography as described [13], [20].

### Protein purification and serum adsorption with magnetic beads

For protein purification of histidine-tagged proteins and analyses of interacting serum proteins, magnetic beads (Dynabeads TALON, Invitrogen Dynal AS, Oslo, Norway) coated with cobalt ions were used. For purification of CRASP-3 or CRASP-5-bound human serum proteins, *E. coli* lysates (1 ml) containing expressed histidine-tagged proteins were incubated with 2 mg of magnetic beads for 10 min at room temperature as recommend by the manufacturer. After four washes with phosphate buffer (50 mM phosphate, 300 mM NaCl, 0.01% Tween20), histidine-tagged proteins coupled onto beads were incubated with NHS for 1 h at room temperature. After extensive washing with phosphate buffer, bound proteins were eluted with 100 mM glycine-HCl (pH 2.0) for 15 min. The eluate and the last wash fraction were separated by 12.5% Glycine-SDS-PAGE under non-reducing conditions followed by staining with silver or Coomassie brilliant blue.

### Sample preparation for mass spectrometry

The selected protein spots were cored from gels and subjected to in-gel digestion protocols as described [67], [68], which were adapted for use on a Microlab® Star digestion robot (Hamilton, Bonaduz, Switzerland). After 12 h, the supernatant was removed and the remaining peptides were extracted three times with 50% (v/v) ACN/5% FA. All fractions were pooled and dried prior to analysis. For MALDI mass spectrometric analysis the samples were solved in 5 µl 50% ACN/1% (v/v) TFA (Fluka, Buchs, Switzerland). 0.5 µl of the sample was mixed with 0.5 µl of matrix (2 mg/ml α-cyano-4-hydroxycinnamic acid (Bruker, Bremen, Germany) in 50% ACN/0.5% (v/v) TFA) directly on a stainless steel MALDI target (Applied Biosystems (ABI), Darmstadt, Germany) and dried under ambient conditions.

### MALDI TOF MS

Delayed extraction™ (DE) MALDI time of flight (TOF) mass spectra were recorded on a Voyager-DE STR instrument (ABI) using a nitrogen laser (λ = 336 nm, repetition rate  = 20 Hz) for desorption and ionisation with an acquisition mass range from 600 m/z to 5000 m/z and the low mass gate set to 550 m/z. The total acceleration voltage was 20 kV with 68.5% grid voltage on the first grid, 0.02% guide wire voltage, 150 ns delay and a mirror voltage ratio of 1.12. Spectra were externally calibrated with Sequazyme™ Peptide Mass Standards Kit (ABI). Between 1000 and 2000 laser shots were accumulated for each mass spectrum. All spectra were smoothed, noise-filtered and deisotoped using Data Explorer (Ver. 4.3, ABI). Deisotoped peaks were labelled by the software and the 100 most intense peaks were used for database searching. Autolytic tryptic peptides or peptides resulting from the identified protein were used for internal calibration.

### Protein database queries

Proteins were identified using Spectrum Mill (Ver. 3.0, Agilent Technologies, Waldbronn, Germany) installed on a local server using the MASCOT (TM) Search Engine, Matrix Science (http://www.matrixscience.com). For PMF data, the 100 most intense peaks were submitted to Spectrum Mill using a search tolerance of 25 ppm.

### Construction of shuttle vectors

Vector pKFSS1, a streptomycin resistant derivative of pBSV2 [69] was used to generate shuttle vectors to allow expression of CRASP-3 or CRASP-5 by the serum-sensitive *B. garinii* strain G1. The *erpP* and *erpA* genes, plus their native promotor regions, were amplified from *B. burgdorferi* s.s strain LW2 by PCR using primers containing respective restriction sites (Table 1). The sequences of the two *erp* genes of strain LW2 is identical to those of *B. burgdorferi* type strain B31. Amplicons generated were digested with BamHI and HindIII and cloned into pKFSS1 at the corresponding restriction sites yielding shuttle vectors pCRASP-3 (expression of CRASP-3) and pCRASP-5 (expression of CRASP-5). Purified recombinant plasmid inserts were subjected to nucleotide sequencing to verify that no mutations had been introduced during PCR and cloning procedures.

**Table 1:** Oligonucleotides used in this study.

Oligonucleotide	Sequence (5′ to 3′)^a^	Use in this work
ErpA(+)	GCATTTGCAATGGATCCATTTTGGGGAGTTG	cloning of *erpA* or *erpP* in pKFSS1
ErpA Hind(-)	CATAATTCTTACAAGAAAGCTTCAGCGCAAAAACTGCAC	cloning of *erpA* in pKFSS1
ErpP Hind(-)	CAGCACAAACAATCAAAGCTTTTTTATTCATAATTATTC	cloning of *erpP* in pKFSS1
CRASP-3 79(+)	GATGAGCCAAAGTAGTGGTGAGATAAACC	amplification of *erpP* gene
CRASP-3 520(-)	CTATTTTAAATTTTTTTTGGATCCTTATTATGGTTTGCATA	amplification of *erpP* gene
CRASP-5 79(+)	GATGAGCAAAGCAATGGAGAGGTAAAGGTC	amplification of *erpA* gene
ErpA 3nc (-)	GTTTTTTTATTCATATACGGGCCCTCCTATATTTCTAAC	amplification of *erpA* gene
aadA+NdeI	CATATGAGGGAAGCGGTGATC	amplification of *aadA* gene
aadR+AatII	GACGTCATTATTTGCCGACTACC	amplification of *aadA* gene
Fla6	AACACACCAGCATCGCTTTCAGGGTCT	amplification of *flaB* gene
Fla7	TATAGATTCAAGTCTATTTTGGAAAGCACCTA	amplification of *flaB* gene

a sequences of specific restriction endonuclease recognition sites are underlined.

### Transformation of serum-sensitive B. garinii

The non-infectious, serum-sensitive *B. garinii* strain G1 was grown in BSK medium and harvested at mid exponential phase (5×10^7^ to 1×10^8^ cells/ml). Electrocompetent cells were prepared as described previously [70] with slight modifications. Briefly, 60 µl aliquots of competent G1 cells were electroporated at 2.5 kV/cm for 4 sec in 2-mm cuvettes with 25 µg of plasmid DNA. Bacteria were transformed with either pCRASP-3, pCRASP-5 or empty pKFSS1. Cells were immediately diluted into 10 ml BSK medium and incubated without antibiotic selection at 33°C for 48 h. For selection of transformants, cells were diluted into 100 ml BSK medium containing 20 µg/ml streptomycin, then 200 µl aliquots were transferred into 96-well plates (Corning). After four to six weeks incubation at 33°C, wells were evaluated for growth by color change of the medium and by dark-field microscopy for the presence of motile spirochetes. Several clones were expanded in 1 ml fresh BSK medium containing streptomycin (20 µg/ml) for 7 to 14 days. Transformed bacteria were then maintained in BSK medium containing 20 µg/ml streptomycin.

### PCR analysis of transformed borrelial cells

Streptomycin-resistant clones of transformed *B. garinii* were characterized by PCR amplification of the introduced *erpP* or *erpA* genes, and the recombinant plasmids' streptomycin resistance gene (*aadA*) using specific primers (Table 1). The native *B. garinii flaB* gene was also PCR amplified as a positive control. Ten microliter aliquots of bacterial cultures grown to mid-exponential phase were used for direct PCR. PCR was carried out for 25 cycles using the following parameters: denaturation at 94°C for 1 min, annealing at 50°C for 1 min, and extension at 72°C for 1 min. Reaction products were separated by agarose gel electrophoresis, and DNA was visualized by ethidium bromide staining and ultraviolet light.

### SDS-PAGE, Western blot and ligand affinity blot analysis

Bacterial cell lysates were subjected to 10% Tricine-SDS-PAGE under reducing conditions and samples obtained by serum adsorption (last wash and elution fractions) were separated by Glycine-SDS-PAGE under non-reducing conditions as previously described [11], [23]. Briefly, after transfer of proteins onto nitrocellulose, nonspecific binding sides were blocked using 5% (w/v) dried milk in TBS (50 mM Tris-HCl pH 7.4, 200 mM NaCl) containing 0.1% Tween20 for 1 h at room temperature. Subsequently, membranes were rinsed four times in TBS containing 0.1% Tween20.

For ligand affinity blot analysis, membranes were incubated for 1 h with NHS. After four washings with TBS containing 0.2% Tween20, membranes were incubated for 1 h with a polyclonal rabbit antiserum that recognizes the amino-terminal regions of CFH, monoclonal antibody (mAb) VIG8 which recognizes the C-terminus of both CFH and CFHR1, or mAb JHD 7.10 which recognizes CFHR1 but not CFH [20], [41]. Following four washes with TBS containing 0.2% Tween20, membranes were incubated with an appropriate peroxidase-conjugated secondary antibody for 1 h. Detection of bound proteins was performed using 3,3′,5,5′-tetramethylbenzidine (TMB) as substrate.

For Western blot analysis, membranes were incubated for 1 h at room temperature with either mAb recognizing CRASP-3/ErpP (N38 1.1), CRASP-5/ErpA (B11), CFH (VIG8), CFHR1 (JHD 7.10), or FlaB (L41 1C11). Following four washes with TBS containing 0.2% Tween20, membranes were probed with peroxidase-conjugated anti-mouse or anti-rabbit secondary antibody (Dako, Glostrup, Denmark) for 60 min at room temperature and bound antibodies were detected using TMB.

### ELISA

Microtiter plates (Nunc-Immuno Module) were coated with CRASP-3 (5 µg/ml) or CRASP-5 (5 µg/ml) over night at 4°C. The plates were washed with PBS containing 0.1% Tween20 and treated for 1 h at RT with blocking buffer (Applichem GmBH, Darmstadt, Germany). After washing, equimolar amounts (33 µM) of CFH, CFHR1, CFHR2 or CFHR5 were added and incubated for 1 h at RT. Thereafter, the wells were washed and bound CFH or CFHR proteins were detected with either goat CFH polyclonal antiserum or mouse CFHR1 monoclonal antiserum (JHD 7.10), which reacts with all the three CFHRs [20]. After washing, protein complexes were identified using appropriate secondary horseradish peroxidase-coupled antiserum. The reaction was developed with 1,2-phenylenediamine dihydrochloride (OPD, DakoCytomation, Glostrup, Denmark) and the absorbance was measured at 490 nm.

### In situ protease accessibility experiments

Viable borreliae were gently washed and resuspended in 500 µl PBS to obtained a density of 8×10^5^/µl. Subsequently, proteinase K in distilled water (Sigma-Aldrich, Deisenhofen, Germany) was added to a final concentration of 100 µg/ml. As a control, intact spirochetes without treatment of protease were also used. Following incubation for 2 h at room temperature, proteinase K was terminated by adding phenylmethylsulfonyl fluoride (Sigma-Aldrich) (50 mg/ml in isopropanol). Cells were then washed gently twice with PBS/5 mM MgCl_2_, resuspended in 20 µl of the same buffer, then lysed by sonication 5 times for 30 sec using a Branson B-12 sonifier (Heinemann, Schwäbisch Gmünd, Germany). Aliquots were separated using Tricine-SDS-PAGE as described above.

### Serum adsorption assay

Borreliae (1×10^9^ cells) grown to mid-log phase were washed with veronal buffered saline and subsequently resuspended in 750 µM NHS supplemented with 34 mM EDTA (pH 8.0) to avoid complement activation. After 1 h incubation at room temperature and four washes with PBSA (0.15 M NaCl, 0.03 M phosphate, 0.02% sodium azide, pH 7.2) containing 0.05% Tween20, proteins bound to the cells surface were eluted with 100 mM glycine-HCl (pH 2.0) for 15 min. Cells were removed by centrifugation at 14,000×g for 10 min at 4°C, and the supernatant and the last wash were separated by Glycine-SDS-PAGE under non-reducing conditions and analyzed by Western blotting as described above.

For experiments using increasing salt concentrations, cells were washed four times with PBSA, sedimented by centrifugation, and resuspended in PBSA containing 450 mM of NaCl. The spirochetes were then incubated for 15 min at room temperature, sedimented and resuspended in PBSA containing the respective concentration of NaCl (750 mM to 1350 mM). This procedure was repeated for each incubation reaction. Finally, bound serum proteins were eluted with 100 mM glycine-HCl (pH 2.0) for 15 min and all supernatants collected (last wash fraction, fractions from the incubation reactions with increasing salt concentrations, and the eluate fraction) were then analyzed by Western blotting using a polyclonal anti-CFHR1 antiserum as described above.

### C3b inactivation assay

The C3b cleavage capacity of *B. garinii* transformants was assayed after incubation of spirochetes (4×10^7^) with PBS supplemented with 750 ng/ml purified CFH for 60 min at room temperature (Calbiochem, Darmstadt, Germany) as described previously [43]. After washing with PBS, cells were resuspended in 50 µl PBS containing 10 ng/ml C3b (Calbiochem) and 20 ng/ml factor I (Calbiochem) and incubated for 60 min at 37°C. Cells were sedimented and the supernatants were mixed with sample buffer. The samples were then subjected to Glycine-SDS-PAGE under reducing conditions and transferred onto a nitrocellulose membrane. C3b degradation products were detected by using polyclonal goat anti-C3 IgG at a final dilution of 1/2000 (Calbiochem) and a secondary peroxidase-conjugated anti-goat IgG antibody (DakoCytomation, Glostrup, Denmark). For detection, 3,3′,5,5′-Tetramethylbenzidine was used as substrate.

### Serum susceptibility testing

Serum susceptibilities of *B. garinii* isolate G1, G1/pKFSS1, G1/pCRASP-3 and G1/pCRASP-5 were assessed by growth inhibition assay. Aliquots (1.25×10^7^ cells) of highly motile spirochetes were diluted into final volumes of 100 µl fresh BSK-medium, which contains 240 µg/ml phenol red. As bacteria grow in BSK, the medium acidifies and the pH indicator dye turns from red to yellow. To each aliquot of bacteria was added either 100 µl NHS or 100 µl heat-inactivated NHS. Bacteria were then held in 96-well microtiter plates for 8 days at 33°C. For controls, aliquots of bacteria were also incubated with 100 µl BSK medium instead of human serum. Bacterial growth was monitored daily by measuring the ratio of culture medium absorbance at 562 versus 630 nm using an ELISA reader (PowerWave 200; Bio-Tek Instruments, Winooski, VT). For calculation of the growth curves the Mikrowin Version 3.0 software (Mikrotek, Overath, Germany) was used. Each experiment was conducted at least three times and means ± SD were calculated.

### Flow cytometry

To analyze binding of CFH to transformed borreliae by flow cytometry 2×10^8^ cells grown to mid-log phase were washed with PBS and subsequently incubated in 300 µl PBS containing 4 µg purified CFH (Calbiochem) for 60 min at room temperature. Afterwards, spirochetes were washed twice and then stained with a monoclonal anti-CFH antibody (Quidel) for 30 min at 4°C, followed by incubation with phycoerythrin-labeld secondary antibody (R&D, Wiesbaden, Germany) for 30 min at 4°C. Samples were analysed immediately on a FACSCalibur (BD, Heidelberg, Germany) using CellQuest Pro software (BD, Heidelberg, Germany).

### Immunofluorescence assay

Spirochetes grown to mid-exponential phase were harvest by centrifugation (5000×g, 30 min), washed, and resuspended in veronal buffered saline (VBS, supplemented with 1 mM Mg^2+^, 0.15 mM Ca^2+^, 0.1% gelatin, pH 7.4).

For detection of deposited complement components on the bacterial surface, spirochetes (6×10^6^) were incubated in 25% NHS and, as a control, in 25% heat-inactivated NHS for 30 min at 37°C with gently agitation. After two washes with PBS containing 0.2% BSA, 10 µl aliquots of cell suspensions were spotted on glass slides and allowed to air dry overnight. Slides were then fixed in methanol for 10 min and air dried for 1 h. After 1 h incubation at 37°C with polyclonal antibodies directed against the complement components C3 (1/500 dilution) (Calbiochem) or C6 (1/100 dilution) (Calbiochem) or a 1/50 dilution of a mAb directed against C5b-9 (Quidel, San Diego, CA, USA) in a humidified chamber, slides were gently washed four times with PBS and subsequently incubated with a 1/2000 dilution of Alexa 488-conjugated anti-goat antibody or Alexa 488-conjugated anti-mouse antibody (Molecular Probes) for 1 h at 37°C.

For detecting surface-exposed proteins, polyclonal rabbit anti-CRASP-3 antiserum (1/50 dilution) or polyclonal rabbit anti-CRASP-5 antiserum (1/20 dilution) was added to the cells for 1 h at 37°C with gently agitation. After two washes with PBS containing 1% BSA, 10 µl aliquots of the cell suspensions were spotted on glass slides and allowed to air dry overnight ( =  unfixed cells). Slides were then fixed in methanol for 10 min, air dried for 1 h following the incubation with an adequate Alexa 488-conjugated antibody. Slides were then gently washed four times with PBS and mounted on ProLong Gold antifade reagent (Molecular Probes) containing DAPI before being sealed. Slides were visualized at a magnification of ×1000 using a Olympus CX40 fluorescence microscope mounted with a DS-5Mc charge-coupled device camera (Nikon).

As a control, periplasmic FlaB was also investigated using unfixed as well as fixed spirochetes as described previously [32]. Briefly, cells were first incubated with a 1/50 dilution of mAb L41 1C11 recognizing FlaB and washed twice with PBS containing 1% BSA ( =  unfixed cells). Aliqouts of 10 µl were then spotted onto glass slides and after air drying at room temperature, cells were fixed with methanol. Slides were incubated with an anti-mouse Alexa 488-conjugated antibody (1/1000 dilution), washed gently four times with PBS, and mounted on ProLong Gold antifade reagent (Molecular Probes) containing DAPI before being sealed. At the same time, spirochetes were first fixed onto coverslips using methanol and then incubated with a 1/50 dilution of mAb L41 1C11 to detected the periplasmic-located FlaB protein ( =  fixed cells). The slides were then proceed as described above.
